# Multi-objective optimization in population pharmacokinetic model selection and optimization: application of NSGA-II in pyDarwin

**DOI:** 10.1007/s10928-026-10036-9

**Published:** 2026-05-16

**Authors:** Xinnong Li, Mark Sale, James Craig, Keith Nieforth, Alex Mazur, Robert R Bies

**Affiliations:** 1 Department of Pharmaceutical Sciences, University at Buffalo, 118 Pharmacy Building, Buffalo, NY 14214 USA; 2 Certara, Radnor, PA 19087 USA

**Keywords:** Machine learning, Pharmacokinetics, Modeling, Multi-objective optimization, Genetic algorithm

## Abstract

The selection of a “good” model usually involves a combination of objective and subjective criteria. Although many aspects of model quality can be expressed numerically, certain desirable characteristics remain difficult—or even impossible—to quantify precisely. Multi-objective optimization (MOO) provides a systematic way to handle this challenge by explicitly incorporating and balancing both objective (measurable) and subjective (judgment-based) considerations when choosing among candidate solutions. The generated Pareto front represents a set of non-dominated models where no single solution can be improved in one objective without sacrificing the performance in another objective. Using the non-dominated sorting genetic algorithm II (NSGA-II), an implementation of MOO, we simultaneously considered objective function value and number of estimated parameters as competing criteria. Concentration measurements of 17-DMAG, quetiapine, clozapine and ziprasidone were applied to build population pharmacokinetic models through traditional stepwise search, machine learning based single-objective hybrid genetic algorithm (SOHGA) and MOO. Local downhill search with MOO was also assessed in this study. While both objectives improved, models with lower objective function value generally contained more estimated parameters. The number of non-dominated solutions for DMAG, ziprasidone, clozapine, and quetiapine was 17, 9, 9, and 13, respectively. The optimal model selected by SOHGA appeared on the Pareto front for DMAG, ziprasidone and clozapine datasets. Overall, MOO provides objective transparency to the cost of tradeoffs between competing model objectives, allowing researchers to better contextualize subjective criteria (e.g., biological plausibility, improvements in diagnostic plots) when aligning model selection with clinical context.

**Supplementary Information:**

The online version contains supplementary material available at 10.1007/s10928-026-10036-9.

## Introduction

Population pharmacokinetic (popPK) modeling is an important tool for characterizing drug absorption, distribution, metabolism, and excretion profiles and identifying clinically relevant covariates that contribute to the variability within a population [1]. Traditionally, model development follows the stepwise procedure (forward addition/backward elimination), which means the model search usually starts from a simple, base model, then iteratively tests hypotheses based on changes in the objective function value (OFV) and pre-specified significance thresholds. While the stepwise search is widely used, these approaches have fundamental limitations. Firstly, the “trivial” model where the search starts is likely very different from the true optimal model, and may result in identifying local minima in the model structure search space. Secondly, forward addition and backward elimination procedure, which is also classified as a “greedy algorithm”, has the risk of missing interaction between model features, such as compartment number, covariate effect, and variability model, as typically one feature is tested at a time [2]. These limitations will make the traditional stepwise search prone to identifying local optima, therefore missing the true optimal model. Thus, alternative, equally valid, and potentially better models may remain undiscovered using the stepwise approach [3, 4]. It is not unexpected that the final model structure identified using a stepwise search may not align with those identified through machine learning (ML)-based global search.

To address these limitations, machine learning-based optimization approaches have been applied to popPK model development [5]. Other existing automated model selection tools include pyDarwin [6], Pharmpy [7], and mlxModelFinder [8]. Pharmpy provides automated workflow in both stepwise search and algorithm-based model structure search. The current implemented algorithms include exhaustive, exhaustive stepwise, and reduced stepwise. The model candidates are compared and ranked using OFV, Akaike Information Criterion (AIC), or Bayesian Information Criterion (BIC). The mlxModelFinder is a package included in MonolixSuite. The algorithm options include decision tree, ant colony optimization, and exhaustive search. The default metric is Bayesian Information Criterion corrected (BICc). When the inter-individual variability is considered in the search space, the evaluation metrics becomes BICc plus a penalty for unstable parameter estimates. Other metric functions can be customized and defined by users. While these two tools present some useful algorithms and metrics for automated model selection, no multi-objective approach generating Pareto optimal solutions has been explored. In pyDarwin, to build a popPK or pharmacodynamic model, the first step is to generate hypotheses related to model structure and parameter values based on the dataset. The set of hypotheses is regarded as a “search space”, where each structure-related hypothesis consists of several mutually exclusive options. The next step is using ML algorithm to select models by comparing the combination of hypotheses. Currently, there are eight algorithms implemented in pyDarwin [6], the software utilized to carry out both a multi-objective optimization (MOO) and single-objective search. The single-objective search, is driven using a fitness function, composed of the OFV and user-defined penalty terms. The hybrid component of this search combines the global search and local downhill search, increasing the efficiency in locating the best model within a generally good region of the search space [9]. The local search is typically called every 5 generations of global search, by systematically flipping each bit the model binary bitstring from 0 to 1 or from 1 to 0 to explore more possible model structures. This algorithm somewhat resembles the traditional forward addition/backward elimination method, changing one “feature” at a time and testing whether the model improves. An important difference between the local search and traditional forward addition/backward elimination is that all features/bit changes are tested in this way in each downhill step. If more than one feature change results in an improved model, the model with the lowest fitness is progressed to the next downhill step. However, one significant disadvantage of a composite single fitness function is that potentially arbitrary weights will be added to each objective [10]. This is not dissimilar from using an information criterion such as the AIC or BIC, where a single composite value (albeit grounded in number theory) returns a single “best” solution. Other commonly used parsimony penalties, which are more arbitrary include 3.84 point for *p* < 0.05 and 6.63 for *p* < 0.01, with 1 degree of freedom. MOO techniques were recently added to pyDarwin to address this concern. Rather than combining weighted objectives into a single composite function, MOO considers multiple criteria simultaneously without including any (potentially arbitrary) penalties. Moreover, rather than producing a single “best” solution, MOO generates a Pareto front of solutions that presents and quantifies trade-offs among the objectives, such as OFV, number of estimated parameters, and other user-defined metrics across the numerically optimal models. This set of non-dominated models are then presented to the modeler who can further evaluate them using subjective criteria (e.g., biological plausibility, diagnostic plots, clinical relevance) and could provide more useful information regarding final model selection [11]. 

In this study, we applied the single objective and MOO model search strategies to concentration measurements for four compounds: DMAG; clozapine; ziprasidone; and quetiapine. PopPK model search results from published traditional stepwise search, single-objective hybrid genetic algorithm (SOHGA) with one- and two-bit local search, and MOO using non-dominated sorting genetic algorithm II (NSGA-II) with and without local search were compared. Here, II refers to the fact that this is the second version of the NSGA algorithm and considers two objectives simultaneously. We hypothesized that the solution from the SOHGA search would be included in the Pareto front, whereas the stepwise search might identify a model that is not on the Pareto front.

## Methods

### Data

PK models were developed independently using manual, SOHGA, and NSGA-II methods for four compounds: DMAG, quetiapine, clozapine, and ziprasidone [12–16]. A simple protocol and final enrollment statistics were summarized in Table 1; available covariates and their distributions were summarized in Table S1. Details of the collection schedule were described in previous publications [12–17]. Ziprasidone, clozapine, and quetiapine were sparsely sampled, with fewer than three samples collected per individual. The DMAG dataset has relatively rich sampling, with participants having an average of more than 15 samples. All of these manually developed models were identified using a stepwise search approach.

**Table 1:** Dataset sample size in ML search and dose administered

Compound	Number of participants	Number of observations	Dose Administered (Median (range))
Ziprasidone	233	569	80 mg (20–160 mg)
Clozapine	391	1142	300 mg (12.5–800 mg)
DMAG	66	951	33 mg (2.2–413 mg)
Quetiapine	404	943	300 mg (25–800 mg)

### Single-objective hybrid search

Single-objective hybrid search was implemented based on the pyDarwin toolbox for each of the four datasets. The related tutorial and test of implemented machine learning algorithms were published previously [5, 9]. The term “single-objective search” means the conducted ML-based search is driven by one fitness function, which is composed of OFV derived from NONMEM and user-defined penalty terms. The OFV was calculated as $\:OFV=-2logL\left(\theta\:\right)$, where $\:L\left(\theta\:\right)$ represents the likelihood of the data given model parameter $\:\theta\:$. The parameters were estimated using first-order conditional estimation with interaction method. In this study, 10 point penalties were added for each estimated parameter; 100 points were added if: the model convergence or the covariance step did not succeed; if there was high correlation (any of the absolute values of the off-diagonal elements of the estimation correlation matrix were > 0.95); and if a high condition number (greater than 1000) was observed. The term “hybrid” refers to an approach that is a combination of ML-based global search and a local search algorithm. The local downhill search consisted of systematically changing one or two features at a time (corresponding to a one- or two-bit downhill search). When doing the local downhill search, first a one-bit search was performed. In the one-bit local search each bit in the binary representation of the models was flipped from 0 to 1 or 1 to 0, resulting in * n* models, where * n* represents n-bit binary string of model structure. This corresponds (roughly) to changing one feature in the model at a time, e.g. 1 to 2 compartments, with or without lag time, with or without volume as a function of weight, with or without between subject variability on a parameter, initial estimate of a parameter of 1 or 10.

A two-bit search (something that can be selected as an option by the users) was conducted following the one-bit search, where all possible combinations of two-bit changes (i.e., up to two model features) were flipped at a time, generating $\:\frac{n\times\:\left(n-1\right)}{2}$ models, where * n* is the number of bits in the genome. If any model generated using this approach was better (with a lower fitness value), it was recorded and preserved for the continuation of the search. The downhill search process was repeated until no further improvement (i.e., no “better” models) were identified. The local downhill search was implemented every 5 generations for this study (this can be customized by the user). Hyperparameter settings were summarized in Table 2. In this study, the SOHGA was the search algorithm for evaluation. An initial population of candidate models was randomly generated to serve as the first generation of models and the model fitness for each was calculated. Parent models were selected using a tournament selection, where pairs of candidates were compared and the model with lower fitness was chosen for reproduction. Selected parents then underwent crossover and mutation operations to generate a new population of candidate models.

**Table 2:** Hyperparameter setting of machine learning algorithms

Parameter name	Description	Value
Single-objective genetic algorithm		
theta	Penalty for each estimated THETA	10
omega	Penalty for each estimated OMEGA	10
sigma	Penalty for each estimated SIGMA	10
convergence	Penalty for failing to converge	100
covariance	Penalty for failing the covariance step	100
correlation	Penalty for failing correlation test	100
condition_number	Penalty for condition number > 1000	100
population_size	Number of candidate models in each generation	80
num_parallel	Number of models to run in parallel	32
num_niches	Number of niches to be used	2
niche_radius	Minimum number of bits different between niches (niche radius)	2
downhill_period	Number of ML generations to be run between downhill searches	5
num_generations	Number of generations to be run	20
crossover_rate	Probability of any pair of parents undergoing cross over	0.95
elitist_num	number of the best model carried over unchanged to the next generation	4
mutation_rate	probability of any new candidate model undergoing mutation	0.95
Non-dominated sorting genetic algorithm		
population_size	Number of candidate models in each generation	100
num_parallel	Number of models to run in parallel	32
num_niches	Number of niches to be used	2
niche_radius	Minimum number of bits different between niches (niche radius)	2
downhill_period	Number of ML generations to be run between downhill searches	5
num_generations	Number of generations to be run	30
crossover_rate	Probability of any pair of parents undergoing cross over	0.95
elitist_num	number of the best model carried over unchanged to the next generation	4
mutation_rate	probability of any new candidate model undergoing mutation	0.95
Effect_limit	Number of effects added to the model	4/6*

^*^4 for ziprasidone and clozapine datasets, and 6 for quetiapine dataset and DMAG downhill search

### Multi-objective search

MOO is an area where the decision-making involves more than one objective function that needs to be solved simultaneously. Normally, these objectives are in conflict and/or compete with each other. The MOO identifies a Pareto front, which is a set of solutions where no single objective can be improved without worsening another objective. The non-dominated models on the Pareto front also represent the best possible trade-offs between competing goals, and one cannot find better solutions within the entire search space. As the multi-objective search proceeds, more feasible model structures within the search space are explored. Generally, the Pareto front will shift from less favorable solutions towards a more optimal area of the search space, simultaneously minimizing all objectives.

In this study, MOO was conducted using the NSGA-II. This algorithm was recently added to pyDarwin and focuses on the search with two objectives simultaneously [18]. NSGA-II is an evolutionary algorithm, where the search starts from a random population and utilizes a genetic algorithm approach to facilitate the selection of models as the analysis progresses. In addition to tournament selection, crossover, and mutation, this algorithm also uses non-dominated sorting and crowding distance sorting to proceed non-dominant search. Non-dominated sorting is a process where every model candidate is compared with other model candidates to determine whether this model is dominating another or it’s been dominated by another. For each model candidate, NSGA-II calculates (1) domination count $\:{n}_{p}$, defined as the number of solutions which dominate the solution p, and (2) $\:{S}_{p}$, defined as a set of solutions that the solution * p* dominates. One solution (model) dominates another if it is not worse on all objectives and better on at least one. A solution/model that is not dominated then is one for which no model in the solution space is better on all objectives, and that model is better than all others on at least one objective. All solutions in the first non-dominated front have a domination count ($\:{n}_{p}$) equal to zero. For each solution * p* in this front, NSGA-II visits all members (* q*) identified its dominated set ($\:{S}_{p}$), and reduces their domination counts by one. If the domination counts of any solution becomes zero after this update, it is assigned to the second non-dominated front. This procedure is then repeated iteratively until all solutions in the population are assigned to a corresponding non-dominated front [19]. Crowding distance sorting is the secondary criterion of the non-dominant search, which calculates the distance between a solution and its neighbor and passes solutions with the greatest distance to the next population. This sorting is done in order to maintain the diversity of the Pareto front. The initial distance for each model candidate * i* in non-dominated set * f* is set to 0. For boundary solutions (when one of the objective functions is fully optimized), the crowding distance is set to infinity, ensuring that these fully optimized models are always selected. For all other points, the crowing distance $\:{d}_{i}$ is calculated as:

$$\:{d}_{i}={d}_{i}+\frac{{f}_{m}\left(i+1\right)-{f}_{m}\left(i-1\right)}{{f}_{m}^{max}-{f}_{m}^{min}}$$

Where the * m* represents the user defined objective features [19]. Using these features, the NSGA-II identifies the Pareto front, where no single model is strictly superior across all objectives [19]. 

NSGA-II search is not driven by a single composite fitness function but driven by a set of user-defined objectives. In this study, the two objectives were objective function value and number of estimated parameters (NEP). The OFV calculation and estimation method were the same with single-objective search. Moreover, the same sequence was applied in the multi-objective optimization as with the single objective search: a local downhill search was introduced after ML-based global search in order to explore the local optimal area more thoroughly. And the local downhill search in NSGA-II was driven by OFV and the NEP. To limit the number of effects (e.g., covariate effect in this case), an option of an effect limit was introduced. The number of effects was defined in the tokens set, with one additional parameter estimated as 1 effect. Each token set included an additional token to define the number of effects for the token set. For example, for a token set describing the relationship between weight and clearance, a power model having 1 effect due to the additional THETA being estimated, and no relationship having 0 effect. The effect limit was implemented by changing the probability of selection of an effect such that 80% of generated models had total effect less than or equal to the effect limit set by users, and models exceeding the effect limit were discarded before running, and replaced by a new generated model. Based on the evaluated model features, the effect limit was set to 4 for ziprasidone and clozapine datasets (mainly covariate relationship), and 6 for quetiapine dataset and DMAG downhill search (consider absorption model and between occasion variability). NSGA-II - related hyperparameter settings were summarized in Table 2. The hyperparameter population size and number of generations were increased to ensure a more complete search for the multi-objective search. To better assess model performance and help decision-making, we included convergence and covariance step completion, and the acceptability of correlation matrices and condition number as basic diagnostics. These criteria (convergence, covariance step, correlation, condition number) did not enter explicitly or directly into the NSGA-II model search.

Within each compound, ML-based search was given identical options, which included ADVAN/TRANS structure, inclusion of between-subject variability, covariate search and associated function form, and form of the residual variability. Absorption models were also considered for drugs that are orally administered. As this is a secondary analysis, there is no intent to reproduce the hypothesis generating process. The search space of ML algorithms referred to model structures considered in published stepwise search, as well as the real datasets we used. The search space for each compound was summarized in Table 3. The final manual and SOHGA models will be compared with the generated non-dominated models on the Pareto front from NSGA-II search.

**Table 3:** Search space applied in machine learning search

Compound	Searched covariates	Compartment	BSV*	RUV*	Others
Ziprasidone	Continuous: age, weight Discrete: sex, race, concomitant medication	1, 2	CL, V, Q2, V2, Ka	Comb, add, prop	Absorption lag
Clozapine	Continuous: age, weight, height Discrete: sex, dosage form	1, 2	CL, V, Q2, V2	Comb, add, prop	NA
DMAG	Continuous: age, weight, creatinine Discrete: sex	1, 2, 3	CL, V, Q2, V2, Q3, V3	Comb, add, prop	BOV on V, CL, Q2
Quetiapine	Continuous: age, weight, Discrete: sex, smoking status	1, 2, 3	CL, V, Q2, V2,	Comb, add, prop	Absorption model

*BSV: between-subject variability; RUV: residual unexplained variability

## Subjective comparison

To further illustrate the utility of the NSGA-II, model comparison among the non-dominated models was evaluated by using the prediction-corrected visual predictive check (pcVPC). These VPCs were performed on the DMAG downhill search, using 4 models selected from the final Pareto front with the NEP = 5, 9, 11, 16, and 1000 replicates in each model. Model parameter estimates with uncertainty of each selected models were summarized in Table S2.

## Results

ML-based search results and stepwise search results are summarized in Tables S3-S6 and Table 4, respectively.

**Table 4:** Manual search result from published paper

Compound	NONMEM model structure	Identified BSV	Identified covariates	Others
Ziprasidone	1-compartment	-	-	Proportional error model, fixed first-order absorption
Clozapine	1-compartment	CL, V	age on CL, sex on CL	Additive error model, fixed absorption rate based on formulation, fixed volume
DMAG	3-compartmet	CL, V1, V2, V3, Q3	-	Proportional error model, BOV on Q2, V1
Quetiapine	1-compartment	CL, V	weight on V	Additive error model, fixed absorption rate

*CL: clearance; V: volume of distribution; Q: intercompartment clearance; BOV: between-occasion variability

### Model structures derived from NSGA-II search

**Fig. 1:**
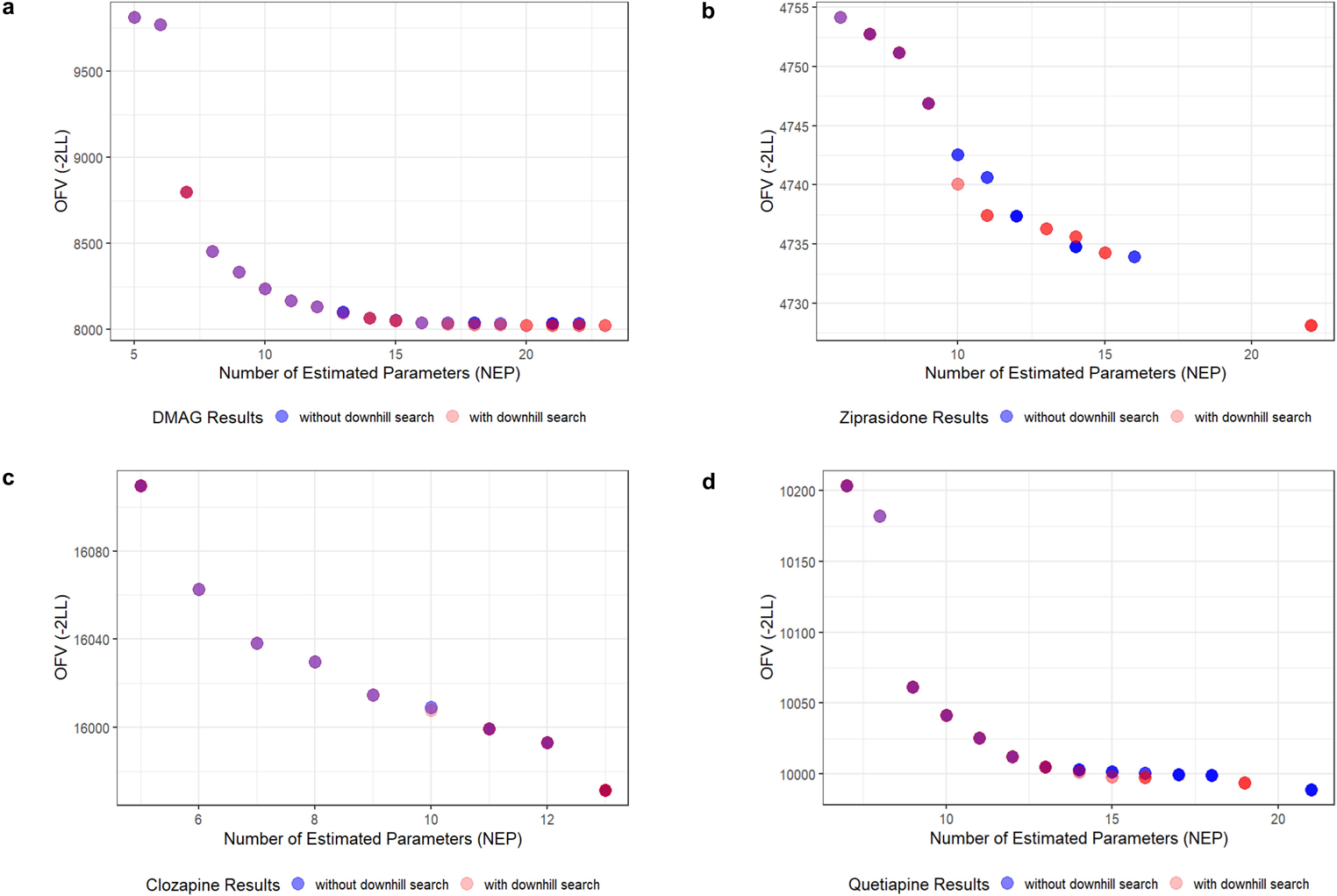
**a-d** Final Pareto fronts of (**a**) DMAG, (**b**) ziprasidone, (**c**) clozapine, (**d**) quetiapine dataset. The blue points represent non-dominated models derived from MOO search without local downhill search. The red points represent non-dominated models derived from MOO search with local downhill search

The Pareto front model solutions are shown in Fig. 1a-d. Each colored point represents one non-dominated model of the final generation, where blue points represent models derived from NSGA-II without downhill search, and red models represent models derived from NSGA-II with downhill search. While both objectives improved, the trade-off between them was clearly observed: models with lower OFV generally contained more estimated parameters. These non-dominated solutions form the Pareto front, where no single solution can outperform all other models across all objectives. These findings highlight that NSGA-II not only recovers known optimal models but also identifies alternative models that represent meaningful trade-offs between fit and complexity.

Tables S3-S6 summarize the non-dominated models identified on the Pareto front in the final generation, representing the optimal solutions within the explored search space. For the DMAG, ziprasidone, and clozapine datasets, the optimal model identified by SOHGA with downhill search was also presented on the Pareto front, confirming the ability of NSGA-II to recover some established solutions.

### Comparison between NSGA-II search with/without local downhill search

For the DMAG dataset, the NEP and OFV range from 5 to 22 and 8034.5-9813.4, respectively for a search without downhill exploration. When the downhill search was incorporated, the range is 5–23 for the NEP, and 8024.6-9813.4 for the OFV. For the ziprasidone dataset, the NEP range is 6–16, and the OFV range is 4733.9-4754.2 without local downhill search, and 6–22 for NEP and 4728.1-4754.2 with local downhill search. For the clozapine dataset, the final Pareto front includes 9 non-dominated models, with NEP numbers ranging from 5 to 13, and OFV values ranging from 15971.6 to 16109.6 for the NSGA-II without the downhill step. The final Pareto front for clozapine includes 10 non-dominated solutions, with NEP from 5 to 13, and OFV from 15971.6 to 16109.6 when the downhill step was utilized. For the quetiapine dataset, without considering local downhill search, there are 13 Pareto optimal solutions, with NEP from 7 to 21, and OFV from 9988.9 to 10203.5. With the local downhill search, the NEP range is from 7 to 19, and OFV from 9993.7 to 10203.5. The different non-dominated models after including local downhill search are labeled in light orange in Tables S3-S6.

### Comparison of the NSGA-II to the SOHGA results and the published popPK models

The DMAG dataset has been extensively investigated in our previously published work on single-objective hybrid search, where ML methods were shown to reliably identify the global optimum searched by exhaustive search. For the traditional stepwise search, a three-compartment model with first-order elimination and proportional error model best described the DMAG dataset. No covariates were selected. The optimal model searched by SOHGA is a three-compartment model, with weight identified as a covariate on volume of distribution and a combined error model. Similar to the stepwise popPK model, many non-dominated models in the NSGA-II search did not include covariates. However, NSGA-II provided additional insights, such as the effect of weight on volume, which was also confirmed by SOHGA and exhaustive search [5, 13]. 

For ziprasidone, the published popPK model is a one-compartment model with first-order absorption and elimination, with between-subject variability (BSV) added on clearance and volume of distribution and a proportional error model. No covariates had a significant effect on the estimated PK parameters. The first-order absorption rate was fixed at 0.5 h⁻¹ in the published model due to instability [16]. By using the SOHGA, the optimal model allowed Ka to be estimated at 0.424 h⁻¹ under the same fixed-effect structure and BSV implementation, with the error model selected as an exponential model. Similar to the stepwise search, no covariate was identified. In the NSGA-II results, the optimal SOHGA model appeared on the final Pareto front. Among the other non-dominated models that passed key diagnostic criteria, covariates age and sex appeared multiple times.

In the clozapine dataset, the published model is a one-compartment model, with sex and age identified as covariates on clearance. First-order absorption rates of two formulations were fixed as 0.8 h⁻¹ and 20 h⁻¹, respectively, and the volume of distribution was fixed as 950 L in the published model [15]. The optimal model from SOHGA search is a one-compartment model with the identified covariate relationship between clearance and sex. Two first-order absorption rate constants were fixed at the same values in the stepwise search due to the instability, while the volume was estimated as 1050 L. In the MOO search, the absorption rates were fixed to the published values, and the volume of distribution value was estimated to fall between 1030 and 1080 L for the models that passed basic diagnostic criteria. Based on the non-dominated models, the covariate relationship between sex and clearance appeared in every non-dominated model on the Pareto front.

By using the stepwise search, the popPK model of quetiapine dataset from the Clinical Antipsychotic Trials of Intervention Effectiveness (CATIE) study was reported to be a one-compartment model, with weight identified as an important covariate on the volume of distribution. The first-order absorption rate was fixed as 2 h⁻¹ because of sparse post-dosing and parameter instability [14]. The optimal model searched by SOHGA is a two-compartment model with first-order absorption and elimination, with identified covariate relationships between clearance and weight and age, and the presence of absorption lag time. Similar to the SOHGA, the NSGA-II estimated the first-order absorption rate constant and identified effects of age on CL and lag time on the absorption process, which may have clinical relevance. The covariate relationship between weight on volume also appeared several times, although the importance was compromised in these complex models that risked overparameterization.

### Subjective comparison among the non-dominated models

**Fig. 2:**
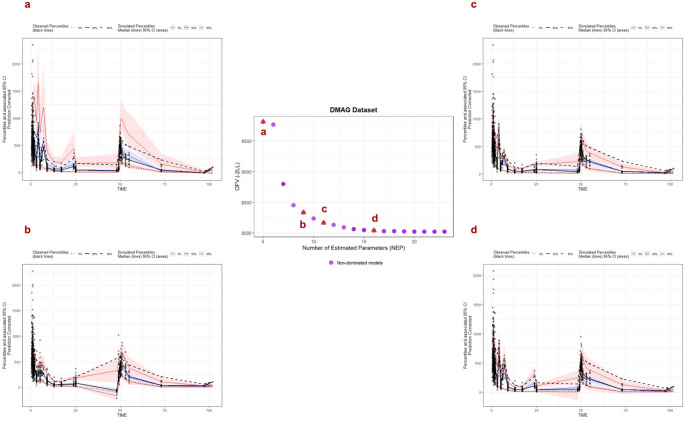
**a-d** Prediction-corrected visual predicted check plots of non-dominated models from DMAG downhill search. **a**, **b**, **c**, **d** represent models with number of estimated parameters (NEP) = 5, 9, 11, 16, respectively

Four non-dominated models that passed all basic diagnostic criteria were selected to perform pcVPCs. The final Pareto front is illustrated in the center panel, where non-dominated models are indicated by purple circles, with x and y axis representing the number of estimated parameters and objective function value. Figure 2a-d correspond to the models with NEP = 5, 9, 11, 16, respectively. In the pcVPC plots, the solid black line is the median of the observed data, the lower and upper dashed black lines are 5th and 95th of the observed data. The solid blue line is the median of the simulated data, the upper and lower red line represent upper 5th and 95th of simulations. The shaded area is the 90% confidence interval (CI). Specific model estimates and uncertainty in parameter estimates for each of these models are shown in Table S2.

The pcVPC results demonstrated a progressive improvement with increasing model complexity, shown by narrower and reduced bias of upper and lower intervals. Figure 2a (NEP = 5, one-compartment model) shows a large deviation between the model and data in the upper and lower prediction intervals. A significant improvement is shown in Fig. 2b (NEP = 9, two-compartment model), especially for the data within the first 24 h. However, the model structure does not appropriately describe the observed data between 25 and 50 h. Figure 2c and d (NEP = 11, 16; three-compartment model) illustrate continued improvement. Compared with the model with NEP = 11, the model structure with NEP = 16 better captures the observations around 25 h. Importantly, the median observations can be predicted reasonably well across all non-dominated models.

In addition to pcVPC, the selected models were further evaluated based on parameter estimates and uncertainty (Table S2). Clearance estimates were consistent across the chosen models, ranging from 8.41 to 9.65 L/hr, with low uncertainty (5–6% RSE). Changing from a two- to a three-compartment structure (model b to c) did not have big impact on the uncertainty. When the model is getting increasingly complex (from model c to d), the overall uncertainty remained low for most estimated parameters, except between subject variability and between occasion variability on central volume, and the power relationship between weight and central volume, which showed higher uncertainty (> 20% RSE).

### Algorithm efficiency comparison

For each dataset, the search space, number of models run in the search, total elapsed time and computational efficiency (time cost for running one model) are summarized in Table S7 for both NSGA-II and SOHGA algorithms with downhill search. The number of models that were run during the search is similar for both algorithms within each dataset, with NSGA-II generally evaluated slightly more models. The total elapsed time increased with more models evaluated, while computational efficiency is comparable for both algorithms. SOHGA showed slightly higher efficiency than NSGA-II, although the difference was minimal for most datasets.

## Discussion

MOO addresses problems where multiple, often conflicting and competing, objectives need to be optimized simultaneously. It has been applied in multiple fields, such as engineering and economics, but has not been widely used in PK/PD model development. Unlike single-objective optimization, which aims for a single best solution, MOO seeks a set of solutions that represent optimal trade-offs between the various objectives [10]. 

In this study, we showed that MOO can be successfully used in selecting population pharmacokinetic models by using NSGA-II and optimizing OFV and NEP simultaneously. To better interpret the results, we incorporated basic model diagnostic criteria at the same time. For example, in the DMAG dataset, the weight effect on volume of distribution and intercompartment clearance, as well as the relationship between creatine clearance and drug clearance should be evaluated further since these covariate relationships appear in the Pareto optimal models that pass all basic diagnostics. For clozapine, the impact of sex on clearance should be considered. Similarly, users may want to focus on the age effect on CL as well as the lag time in absorption for quetiapine. The age, sex, and weight effects on volume of distribution as well as the impact of age on absorption rate and clearance may be important for ziprasidone given the results on the Pareto front. While these diagnostics can provide an initial filter, they are not sufficient for final decision-making. Users are recommended to further evaluate these models using diagnostic plots and clinical plausibility.

As a representative subjective criterion, we compared the pcVPCs of four non-dominated models on the Pareto front from DMAG downhill search. A consistent improvement in the upper and lower predictions was observed when the model structure increases from one to three compartments. Within models sharing the same number of compartments, models that identified covariate effect and included more between-subject variabilities (BSV) and between-occasion variabilities (BOV) showed marginal improvements in specific regions of the data. Across the comparisons we made in this study, a more complex model can better explain the variability. However, all models evaluated here have the same or less complexity compared to the global optimal model derived from the exhaustive search, and all pass the basic diagnostic criteria. Further analysis will be conducted to evaluate if the VPC degrades when the models become overparameterized.

In machine learning based single-objective model building, a local downhill search has been proven necessary to detect the global minima within a search space [5]. By performing a more exhaustive search in a local area, this approach can improve the efficiency of identifying the optimal solution. In this study, we compared the results of search with and without local downhill exploration. MOO with local downhill search generated similar non-dominated models to those identified without local downhill search, especially in more parsimonious models. Under the same level of parsimony, incorporation of the downhill search is more likely, but not guaranteed, to yield models with lower OFV. For example, in the DMAG dataset, without local search, models with 17–18 parameters have OFVs in the 8040–8042 range, whereas with local downhill search, OFV as low as 8029.9 was identified. However, some of these locally searched models may not pass the basic diagnostic criteria (e.g. acceptable condition number).

Similar to other automated methods, MOO does not replace the hypothesis-generating process. That means, the usage of ML algorithm in the popPK model search should be based on thorough data examination and exploration. Moreover, the building of search space should be reasonable and consider the biological plausibility. For example, the drug systemic clearance is not expected to change when the formulation is changed. Therefore, the effect of formulation on clearance should not be included in the search space. At the same time, the final model selection after running the search should not be done blindly without consideration of physiologic, pharmacologic, physicochemical and pharmaceutical factors. In this study, we presented several basic diagnostic metrics such as convergence step, covariance step, correlation metrics, and condition number to serve as an initial step to evaluate model performance. However, additional factors regarding biological plausibility, diagnostic plots, and other subjective criteria should be carefully considered before accepting a model. MOO provides a “short list” of numerically superior models at points of trade-off between competing objectives, revealing inherent compromises in the system and allowing for better informed assessment of non-quantifiable or subjective model selection criteria.

In clinical datasets, the number of potential covariates of interest can be significant (often more than 20 factors), and when combined with other model features such as compartment number and residual error model, can significantly expand the model search space to an intractable level. Incorporating all possible model features in a global search can lead to many problems, including high computational load, much longer search time, risk of overparameterization, and inflated alpha error. When there are too many features, especially true for when covariate effects are included in the final non-dominated models, the process of model selection and interpretation becomes increasingly difficult and unreliable. To address these challenges, we introduced an effect limit hyperparameter.

The effect limit should reflect the expected complexity that the dataset can support. In practice, most datasets support only a limited number of covariate relationships (typically 1–6), and excessive model complexity can lead to instability and overparameterization. The effect limit does not constrain the final model directly, but instead prevents the generation of implausibly complex candidate models during the search, thereby improving computational efficiency and interpretability.

While the present study focuses on two objectives, OFV and model complexity (number of model parameters), both are numerical and statistically defined, and the relationship between them is relatively well understood. Future work could incorporate additional objectives such as estimate precision or clinically relevant endpoints or uncertainty in parameter estimates to fully leverage the potential of multi-objective optimization in model development.

Overall, using single-objective method or multi-objective method is target driven. Users need to decide if they would like a single optimal model (perhaps in later phase, hypothesis confirming analysis) or a set of non-dominated models within the search space, which may be more appropriate for early, exploratory model selection.

## Conclusion

We demonstrated the application of MOO, specifically the use of the NSGA-II algorithm implemented in pyDarwin, as an automated strategy for population PK model search and selection was able to identify a Pareto front of nondominated solutions for the trade-off of number of estimated parameters and objective function value. This enabled the evaluation of trade-offs between fit and complexity. Compared with other model search methods, MOO framework has the advantage of global search, reducing the risk of being trapped in local minima, while avoiding adding potentially arbitrary penalties in a single-objective driven composite function. It can also provide more insights into clinically relevant covariates and alternative model structures of comparable or improved performance. Furthermore, including the local search process in the MOO enables a more thorough exploration of the search space and can identify models with lower OFV, but a careful evaluation is necessary when making decisions about these models.

Overall, stepwise popPK models demonstrate great parsimony, and single-objective search provides one single optimal model across the entire search space. In this study, MOO search not only replicated key clinical relationships identified in the stepwise search, but also revealed alternative covariates. By generating a set of Pareto-optimal solutions, MOO enables researchers to balance fit and complexity while incorporating subjective criteria such as biological plausibility and clinical relevance to accelerate robust model development and clinical decision making. This broader perspective shows the advantage of MOO as a powerful complement to the traditional stepwise approach and single-objective search.

## Electronic Supplementary Material

Below is the link to the electronic supplementary material.

Supplementary Material 1 (PDF 298 KB)

## Data Availability

Datasets were analyzed, no new datasets were generated. CATIE datasets can be requested through: https://nda.nih.gov/nda/access-data-info#getting-started.
