# Supplementary material for: Multi-objective optimization in population pharmacokinetic model selection and optimization: application of NSGA-II in pyDarwin
Source: J Pharmacokinet Pharmacodyn. 2026 May 16;53(4):26. doi: 10.1007/s10928-026-10036-9 (PMC13179877; doi:10.1007/s10928-026-10036-9)
Supplement: Supplementary file 1 — Supplementary Material 1 (PDF 298 KB) [file 10928_2026_10036_MOESM1_ESM.pdf]

Table S1. Covariate distribution of ziprasidone, clozapine, quetiapine and DMAG datasets

Ziprasidone (N = 233)		
	n (%)	Median (range)
Age (yrs)		42 (18-67)
Weight (lbs.)		190 (101-365)
Gender<br>Male<br>Female	 162 (70)<br>71 (30)	
Race<br>Black/African American<br>White<br>Others	 62 (27)<br>158 (68)<br>13 (5)	
Smoking status<br>Active smoker<br>Inactive smoker<br>Nonsmoker	 142 (61)<br>90 (39)<br>1 (0.4)	
Medication<br>1A2 substrate/inhibitor<br>2D6 substrate/inhibitor<br>2C9 substrate/inhibitor<br>2C19 substrate/inhibitor<br>3A4 substrate/inhibitor	 25<br>77<br>38<br>34<br>75	
**Clozapine (N = 391)**		
	n (%)	Median (range)
Age (yrs)		39 (11-79)
Weight (kg)		80 (46-170)
Gender<br>Male<br>Female	 278 (71)<br>113 (29)	
Impatient<br>Outpatient	62 (27)<br>158 (68)	
Formulation<br>Tablet<br>Suspension	 368 (94)<br>23 (6)	
**Quetiapine (N = 404)**		
	n (%)	Median (range)
Age (yrs)		44 (18-94)
Weight (lbs.)		186 (81-367)

Gender<br>Male<br>Female	 283 (70)<br>121 (30)	
Disease<br>Alzheimer's disease<br>Schizophrenia	 79 (34)<br>325 (66)	
Smoking status<br>Active smokers<br>Non-active smokers	 205 (51)<br>199 (49)	
**DMAG (N = 66)**		
	n (%)	Median (range)
Age (yrs)		63 (28-82)
Weight (kg)		80.3 (48.2-136.5)
Blood urea nitrogen (mg/dL)		15 (5-70)
Creatinine (mg/dL)		1 (0.6 -1.8)
Gender<br>Male<br>Female	 41 (62)<br>25 (38)	

Table S2. Model parameter estimates with uncertainty for DMAG models drawn from the Pareto optimal front trading off objective function value and number of estimated parameters

**Model a (NEP = 5, OFV = 9813.4)**			
Parameter	Population estimates (RSE%)	Between subject variability (RSE%)	Between-occasion variability (RSE%)
CL (L/hr)	8.41(5)	35.8% (10)	-
V (L)	98.4 (4)	31.3% (10)	-
Proportional error	0.214 (6)	-	-
**Model b (NEP = 9, OFV = 8335.4)**			
Parameter	Population estimates (RSE%)	Between subject variability (RSE%)	Between-occasion variability (RSE%)
CL (L/hr)	9.65 (6)	43.4% (9)	-
Q (L/hr)	61.8 (6)	-	45.9% (11)
V (L)	37.8 (7)	51.9% (11)	-
V2 (L)	127 (6)	48.4% (10)	-
Proportional error	0.034 (6)	-	-
**Model c (NEP = 11, OFV = 8166.5)**			
Parameter	Population estimates (RSE%)	Between subject variability (RSE%)	Between-occasion variability (RSE%)
CL (L/hr)	9.26 (6)	43.5% (11)	27.7% (12)
Q2 (L/hr)	81.8 (4)	-	-
Q3 (L/hr)	9.47 (8)	-	-
V (L)	28.1(10)	70.2% (11)	-
V2 (L)	75.6 (9)	63.3% (10)	-
V3 (L)	85.3 (7)	-	-
Proportional error	0.029 (6)	-	-
**Model d (NEP = 16, OFV = 8041.3)**			
Parameter	Population estimates (RSE%)	Between subject variability (RSE%)	Between-occasion variability (RSE%)
CL (L/hr)	8.64 (6)	50.3% (11)	30.3% (13)
Q2 (L/hr)	79.3 (4)	-	-
Q3 (L/hr)	9.74 (7)	75.8% (10)	28.6% (13)
V (L)	29.1(7)	42.9% (24)	31.1% (34)
V2 (L)	71.9 (7)	58.7% (11)	-
V3 (L)	136 (7)	-	-

Power relationship between weight and volume	1.31(22)	-	-
Proportional error	0.017(7)	-	-
Additive error (mg/L)	14.7 (36)	-	-

Table S3: NSGA-II and SOHGA search results of DAMG dataset

DMAG NSGA-II (without downhill search)										
Total # of parameter	OFV	COM	BSV	BOV	RUV	Covariate	Convergence	Covariance	Correlation	Condition#
5	9813.4	1	V, CL	-	prop	-	TRUE	TRUE	TRUE	TRUE
6	9769.9	1	V, CL	-	comb	-	TRUE	TRUE	TRUE	TRUE
7	8799.4	2	V, CL	-	prop	-	FALSE	TRUE	TRUE	TRUE
8	8452.4	2	V, CL, V2	-	prop	-	TRUE	TRUE	TRUE	TRUE
9	8335.4	2	V, CL, V2	Q	prop	-	TRUE	TRUE	TRUE	TRUE
10	8238.3	3	V, CL, V2	-	prop	-	TRUE	TRUE	TRUE	TRUE
11	8166.5	3	V, CL, V2	CL	prop	-	TRUE	TRUE	TRUE	TRUE
12	8134.1	3	V, CL, V2	CL, Q2	prop		TRUE	TRUE	TRUE	TRUE
13	8101.6	3	V, CL, Q3, V2	CL	comb		FALSE	TRUE	FALSE	FALSE
14	8065.6	3	V, CL, V2, Q3	CL, Q2	comb		TRUE	TRUE	TRUE	TRUE
15	8056.9	3	V, CL, V2, Q3	V, CL Q2	comb	-	TRUE	TRUE	TRUE	TRUE
16	8041.3	3	V, CL, V2, Q3	V, CL Q2	comb	V~WT	TRUE	TRUE	TRUE	TRUE
17	8041.2	3	V, CL, V2, Q3	V, CL Q2	comb	Q2~WT V~WT	TRUE	TRUE	TRUE	TRUE
18	8040.8	3	V, CL, V2, Q3	V, CL Q2	comb	V3~WT, V~WT V2~WT	FALSE	TRUE	FALSE	FALSE

19	8035.1	3	V, CL, V2, V3, Q3	V, CL Q2	comb	Q~WT, V~WT, CL~SCR	TRUE	TRUE	TRUE	TRUE
21	8034.6	3	V, CL, V2, V3, Q3	V, CL Q2	comb	Q~WT, Q2~WT, V3~WT, V~SEX, CL~SCR	FALSE	TRUE	FALSE	FALSE
22	8034.5	3	V, CL, V2, V3, Q3	V, CL Q2	comb	Q2~WT, V3~WT, V~SEX, CL~SCR, CL~WT, CL~SEX	FALSE	TRUE	TRUE	TRUE
**DMAG NSGA-II (with downhill search)**										
Total # of parameter	OFV	COM	BSV	BOV	RUV	Covariate	Convergence	Covariance	Correlation	Condition#
5	9813.4	1	V, CL	-	prop	-	TRUE	TRUE	TRUE	TRUE
6	9769.9	1	V, CL	-	comb	-	TRUE	TRUE	TRUE	TRUE
7	8799.4	2	V, CL	-	prop	-	FALSE	TRUE	TRUE	TRUE
8	8452.4	2	V, CL, V2	-	prop	-	TRUE	TRUE	TRUE	TRUE
9	8335.4	2	V, CL, V2	Q	prop	-	TRUE	TRUE	TRUE	TRUE
10	8238.3	3	V, CL, V2	-	prop	-	TRUE	TRUE	TRUE	TRUE
11	8166.5	3	V, CL, V2	CL	prop	-	TRUE	TRUE	TRUE	TRUE
12	8134.1	3	V, CL, V2	CL, Q2	prop	-	TRUE	TRUE	TRUE	TRUE
13	8094.8	3	V, CL, V2	CL, Q2	comb	-	TRUE	TRUE	FALSE	FALSE
14	8065.6	3	V, CL, V2, Q3	CL Q2	comb	-	FALSE	TRUE	TRUE	TRUE
15	8050.9	3	V, CL, V2, Q3	CL Q2	comb	V~WT	FALSE	TRUE	TRUE	TRUE

16	8041.3	3	V, CL, V2, Q3	V, CL Q2	comb	V~WT	TRUE	TRUE	TRUE	TRUE
17	8032.7	3	V, CL, V2, V3, Q3	V, CL Q2	comb	V~WT	FALSE	TRUE	FALSE	FALSE
18	8029.9	3	V, CL, V2, V3, Q3	V, CL Q2	comb	V~WT CL~SCR	FALSE	TRUE	FALSE	FALSE
19	8027.4	3	V, CL, V2, V3, Q3	V, CL Q2	comb	V~WT, V~SEX, CL~SCR	TRUE	TRUE	TRUE	TRUE
20	8025.0	3	V, CL, V2, V3, Q3	V, CL Q2	comb	V~WT, V~SEX, CL~SCR, CL~SEX	FALSE	TRUE	FALSE	FALSE
21	8024.8	3	V, CL, V2, V3, Q3	V, CL Q2	comb	V~WT, V~SEX, CL~SCR, CL~SEX, Q2~WT	FALSE	TRUE	FALSE	FALSE
22	8024.7	3	V, CL, V2, V3, Q3	V, CL Q2	comb	V2~WT, V~WT, CL~SCR, CL~SEX, Q2~WT, Q3~WT	FALSE	TRUE	FALSE	FALSE
23	8024.6	3	V, CL, V2, V3, Q3	V, CL Q2	comb	Q2~WT, Q3~WT, V~WT, V~SEX, CL~SCR, CL~WT, CL~SEX	FALSE	TRUE	FALSE	FALSE
**DMAG SOHGA (with downhill search)**										
Total # of parameter	OFV	COM	BSV	BOV	RUV	Covariate	Convergence	Covariance	Correlation	Condition#
16	8041.3	3	V, CL, V2, Q3	V, CL Q2	comb	V~WT	TRUE	TRUE	TRUE	TRUE

Table S4: NSGA-II and SOHGA search results of ziprasidone dataset

Ziprasidone NSGA-II (without downhill search)									
Total # of parameters	OFV	COM	BSV	RUV	Covariate	Convergence	Covariance	Correlation	Condition#
6	4754.2	1	V, CL	prop	-	TRUE	TRUE	TRUE	TRUE
7	4752.8	1	V, CL	prop	CL~AGE	TRUE	TRUE	TRUE	TRUE
8	4751.2	1	V, CL	prop	CL~WT, CL~AGE	FALSE	TRUE	FALSE	FALSE
9	4746.9	1	V, CL,KA	prop	V~AGE, KA~AGE	TRUE	TRUE	TRUE	TRUE
10	4742.5	1	V, CL, KA	prop	V~SEX, V~AGE, KA~AGE	TRUE	TRUE	TRUE	TRUE
11	4740.7	1	V, CL, KA	prop	V~WT, V~SEX, V~AGE, KA~AGE	TRUE	TRUE	TRUE	TRUE
12	4737.4	1	V, CL, KA	prop	CL~RACE, V~AGE, CL~WT, KA~AGE	FALSE	TRUE	TRUE	TRUE
14	4734.6	2	V, CL, V2	comb	CL~CYP, CL~WT, V~WT, Q~WT	FALSE	FALSE	FALSE	FALSE
16	4733.9	2	V, CL, V2, Q	prop	V~WT, V~AGE, CL~CYP, CL~WT KA~AGE ALAG	TRUE	FALSE	FALSE	FALSE
**Ziprasidone NSGA-II (with downhill search)**									
Total # of parameters	OFV	COM	BSV	RUV	Covariate	Convergence	Covariance	Correlation	Condition#
6	4754.2	1	V, CL	prop	-	TRUE	TRUE	TRUE	TRUE
7	4752.8	1	V, CL	prop	CL~AGE	TRUE	TRUE	TRUE	TRUE
8	4751.2	1	V, CL	prop	CL~WT, CL~AGE	FALSE	TRUE	FALSE	FALSE
9	4746.9	1	V, CL,KA	prop	V~AGE, KA~AGE	TRUE	TRUE	TRUE	TRUE
10	4740.1	1	V, CL, KA	prop	V~WT, V~AGE, KA~AGE	TRUE	TRUE	TRUE	TRUE
11	4737.5	1	V, CL, KA	prop	V~WT, V~AGE,	FALSE	FALSE	FALSE	FALSE

					KA~AGE, CL~WT				
13	4736.3	2	V, CL, KA	prop	V~WT, CL~CYP, Q~WT, CL~AGE	FALSE	FALSE	FALSE	FALSE
15	4734.3	2	V, CL, V2, Q, V2	prop	V~SEX, CL~AGE, CL~CYP, CL~WT, KA~AGE	FALSE	TRUE	FALSE	FALSE
22	4728.1	2	V, CL, KA, Q, V2	prop	Q~WT, V~WT, V~SEX CL~AGE, CL~SEX, CL~RACE, CL~CYP, CL~WT, KA~AGE, ALAG	FALSE	FALSE	FALSE	FALSE
**Ziprasidone SOHGA (with downhill search)**									
Total # of parameters	OFV	COM	BSV	RUV	Covariate	Convergence	Covariance	Correlation	Condition#
6	4754.2	1	V, CL	prop	-	TRUE	TRUE	TRUE	TRUE

Table S5: NSGA-II and SOHGA search results of clozapine dataset

**Clozapine NSGA-II (without downhill search)**									
Total # of parameters	OFV	COM	BSV	RUV	Covariate	Convergence	Covariance	Correlation	Condition#
5	16109.6	1	V, CL	prop	-	TRUE	TRUE	TRUE	TRUE
6	16062.6	1	V, CL	comb	-	TRUE	TRUE	TRUE	TRUE
7	16038.2	1	V, CL	comb	CL~SEX	TRUE	TRUE	TRUE	TRUE
8	16029.9	1	V, CL	comb	CL~SEX, CL~WT	TRUE	TRUE	TRUE	TRUE
9	16014.7	2	V, CL	comb	CL~SEX	TRUE	TRUE	TRUE	TRUE
10	16009.0	2	V, CL	comb	CL~WT, CL~SEX	TRUE	TRUE	TRUE	TRUE
11	15999.29	2	V, CL, Q	comb	CL~WT, CL~SEX	FALSE	TRUE	FALSE	FALSE
12	15993.31	2	V, CL, Q	comb	CL~SEX, CL~WT, CL~AGE	FALSE	TRUE	FALSE	FALSE
13	15971.6	2	V, CL	comb	Q~WT, CL~WT, CL~SEX, V~WT, V~SEX	FALSE	FALSE	FALSE	FALSE
**Clozapine NSGA-II (with downhill search)**									
Total # of parameters	OFV	COM	BSV	RUV	Covariate	Convergence	Covariance	Correlation	Condition#
5	16109.6	1	V, CL	prop	-	TRUE	TRUE	TRUE	TRUE
6	16062.6	1	V, CL	comb	-	TRUE	TRUE	TRUE	TRUE
7	16038.2	1	V, CL	comb	CL~SEX	TRUE	TRUE	TRUE	TRUE
8	16029.9	1	V, CL	comb	CL~SEX, CL~WT	TRUE	TRUE	TRUE	TRUE
9	16014.7	2	V, CL	comb	CL~SEX	TRUE	TRUE	TRUE	TRUE
10	16007.8	2	V, CL	comb	CL~SEX CL~AGE	TRUE	TRUE	TRUE	TRUE
11	15999.29	2	V, CL, Q	comb	CL~WT, CL~SEX	FALSE	TRUE	FALSE	FALSE
12	15993.31	2	V, CL, Q	comb	CL~SEX, CL~WT, CL~AGE	FALSE	TRUE	FALSE	FALSE
13	15971.6	2	V, CL	comb	Q~WT, CL~SEX, CL~WT, V~SEX, V~WT	FALSE	FALSE	FALSE	FALSE

**Clozapine SOHGA (with downhill search)**									
Total # of parameters	OFV	COM	BSV	RUV	Covariate	Convergence	Covariance	Correlation	Condition#
7	16038.2	1	V, CL	comb	CL~SEX	TRUE	TRUE	TRUE	TRUE

Table S6: NSGA-II and SOHGA search results of quetiapine dataset

**Quetiapine NSGA-II (without downhill search)**									
Total # of parameters	OFV	COM	BSV	RUV	Covariate	Convergence	Covariance	Correlation	Condition#
7	10203.5	1	V, CL, KA	prop	-	FALSE	TRUE	TRUE	TRUE
8	10182.2	1	V, CL, KA	comb	-	TRUE	TRUE	TRUE	TRUE
9	10061.2	1	V, CL, KA	prop	-	TRUE	TRUE	TRUE	TRUE
10	10041.4	2	V, CL, KA	prop	ALAG	FALSE	TRUE	FALSE	FALSE
11	10025.1	2	V, CL, KA	prop	CL~AGE, ALAG	TRUE	TRUE	TRUE	TRUE
12	10012.2	2	V, CL, KA	prop	V~WT, V~AGE, ALAG	TRUE	TRUE	FALSE	TRUE
13	10004.9	2	V, CL, KA	prop	V~WT, CL~AGE, KA~SMOK, ALAG	FALSE	TRUE	FALSE	FALSE
14	10002.8	2	V, CL, KA, ALAG	prop	V~WT, V~SEX, CL~AGE, KA~SEX, ALAG	FALSE	FALSE	FALSE	FALSE
15	10001.5	2	V, CL, KA, ALAG	prop	V~WT, V~AGE, CL~AGE, KA~SEX, ALAG	FALSE	TRUE	FALSE	FALSE
16	9999.8	2	V, CL, KA, ALAG	prop	V~WT, V~SEX, V~AGE CL~AGE, KA~SEX, ALAG	TRUE	TRUE	TRUE	TRUE
17	9999.3	2	V, CL, KA	prop	V~WT, CL~AGE, V~SEX, CL~AGE, CL~SMOK, KA~SEX,	FALSE	TRUE	FALSE	FALSE

					ALAG, D1				
18	9999.0	2	V, CL, KA, Q, V3	comb	V~WT, CL~AGE, V~SEX, CL~AGE, KA~SEX, ALAG, D1	FALSE	TRUE	FALSE	FALSE
21	9988.9		V, CL, KA, Q, V3	comb	V~WT, V~SEX, V~GE, CL~WT, CL~AGE, KA~SMOK, KA~SEX, AKLG, D1	FALSE	FALSE	FALSE	FALSE
**Quetiapine NSGA-II (with downhill search)**									
Total # of parameters	OFV	COM	BSV	RUV	Covariate	Convergence	Covariance	Correlation	Condition#
7	10203.5	1	V, CL, KA	prop	-	FALSE	TRUE	TRUE	TRUE
8	10182.2	1	V, CL, KA	comb	-	TRUE	TRUE	TRUE	TRUE
9	10061.2	2	V, CL, KA	prop	-	TRUE	TRUE	TRUE	TRUE
10	10041.4	2	V, CL, KA	prop	ALAG	FALSE	TRUE	FALSE	FALSE
11	10025.1	2	V, CL, KA	prop	CL~AGE, ALAG	TRUE	TRUE	TRUE	TRUE
12	10012.2	2	V, CL, KA	prop	V~WT, V~AGE, ALAG	TRUE	TRUE	FALSE	TRUE
13	10004.9	2	V, CL, KA	prop	V~WT, CL~AGE, KA~SMOK, ALAG	FALSE	TRUE	FALSE	FALSE
14	10001.4	2	V, CL, KA	prop	V~WT, V~AGE, CL~WT, CL~AGE, ALAG	FALSE	TRUE	FALSE	FALSE
15	9998.0	2	V, CL, KA	prop	V~WT, V~SEX, V~AGE,	FALSE	FALSE	FALSE	FALSE

					CL~AGE, KA~SEX, ALAG				
16	9997.6	2	V, CL, KA	comb	V~WT, V~AGE CL~AGE, CL~SMOK, KA~SEX, ALAG	FALSE	TRUE	FALSE	FALSE
19	9993.7	2	V, CL, KA	comb	V~WT, V~SEX, CL~WT, CL~SEX, CL~SMOK, CL~AGE, KA~SEX, KA~SMOK ALAG	FALSE	FALSE	FALSE	FALSE
**Quetiapine SOHGA (with downhill search)**									
Total # of parameters	OFV	COM	BSV	RUV	Covariate	Convergence	Covariance	Correlation	Condition#
13	10027.7	2	V, CL, KA, Q	prop	CL~WT, CL~AGE, ALAG	TRUE	TRUE	TRUE	TRUE

*green highlight represents the model structure derived from single-objective hybrid genetic algorithm (SOHGA) also appears on the Pareto front of the non-dominated sorting genetic algorithm search (NSGA-II)

*light orange color highlight represents the model structures searched by NSGA-II combined with local downhill search are different from the model structures derived from NSGA-II without local downhill search under the same parsimony (number of estimated parameters)

Table S7. Summary of algorithm performance of ziprasidone, clozapine, quetiapine and DMAG datasetS

	Search space	Model run in search	Elapsed time (min)	Time (min)/model run in search
**Ziprasidone**				
NSGA-II with downhill	663,552	2013	104	0.05
SOHGA with downhill	663,552	1910	84.3	0.04
**Clozapine**				
NSGA-II with downhill	62,208	1211	111.4	0.09
SOHGA with downhill	62,208	654	64	0.1
**Quetiapine**				
NSGA-II with downhill	829,440	2844	428.6	0.15
SOHGA with downhill	829,440	2227	173.7	0.08
**DMAG**				
NSGA-II with downhill	1,572,864	2191	352.6	0.16
SOHGA with downhill	1,572,864	2393	321.8	0.13

List of Abbreviations:

Abbreviations	Definition
MOO	Multi-objective optimization
SOHGA	Single-objective hybrid genetic algorithm
popPK	Population pharmacokinetic
OFV	Objective function value
ML	Machine learning
NSGA-II	Non-dominated sorting genetic algorithm 2
NEP	Number of estimated parameters
pcVPC	Prediction-corrected visual predictive check
CI	Confidence interval
BSV	Between subject variability
BOV	Between occasion variability
RUV	Residual unexplained variability
